# Macrophage polarization‐related gene signature for risk stratification and prognosis of survival in gliomas

**DOI:** 10.1111/jcmm.70000

**Published:** 2024-10-24

**Authors:** Weiming Zhong, Kaifen Xiong, Shuwang Li, Chuntao Li

**Affiliations:** ^1^ Department of Neurosurgery, Xiangya Hospital Central South University Changsha People's Republic of China; ^2^ Hypothalamic‐Pituitary Research Center, Xiangya Hospital Central South University Changsha Hunan People's Republic of China; ^3^ Department of Dermatology Shenzhen People’s Hospital (The Second Clinical Medical College, Jinan University; The First Affiliated Hospital, Southern University of Science and Technology) Shenzhen Guangdong People's Republic of China; ^4^ Department of Neurosurgery The Second People's Hospital of Hunan Province Changsha People's Republic of China

**Keywords:** glioma, immune infiltration, immunotherapy, macrophage polarization, tumour microenvironment

## Abstract

Macrophage polarization plays an essential role in tumour immune cell infiltration and tumour growth. In this study, we selected a series of genes distinguishing between M1 and M2 macrophages and explored their prognostic value in gliomas. A total of 170 genes were included in our study. The CGGA database was used as the training cohort and the TCGA database as the validation cohort. The biological processes and functions were identified by GO and KEGG analysis. Kaplan–Meier analysis was used to compare survival differences between groups. Importantly, we built a risk score model using Cox regression analysis based on the CGGA and verified it in the TCGA database and our sequencing data. Patients with gliomas in the high‐risk group were associated with high pathologic grade, IDH WT status, MGMT promoter unmethylation, 1p19q non‐codeletion and prone to have a poor outcome. GEPIA results revealed that CD300C, CNRIP1 and MYO1F are the most related genes of immune infiltrations. The differential expression of these genes between low‐grade gliomas and glioblastomas was confirmed by q‐RT‐PCR. Macrophage polarization‐related gene signatures can predict the malignancy and outcome of patients with gliomas and might act as a promising target for glioma immunotherapy in the future.

## 1 INTRODUCTION

Diffuse gliomas are the most common malignant tumours of the central nervous system (CNS) with high neurologic morbidity and mortality, originating from the gluey supportive cells (glial cells) surrounding nerve cells.^1^ More than 100,000 people worldwide are diagnosed with gliomas every year, accounting for nearly 2% of all newly diagnosed cancers and more than 50% of all primary brain tumours.^2^, ^3^ Several factors affect the prognosis of patients with gliomas, including age,^4^ grade,^5^ IDH mutant status,^6^ MGMT promoter methylation status,^7^ 1p19q codeletion status^8^ and glioma stem cell status.^9^ Currently recognized standardized treatment for gliomas include surgery, radiotherapy and chemotherapy with temozolomide (TMZ).^10^ Although the diagnosis and the therapy of the malignancy have improved in recent years, the outcome of patients with gliomas is still unsatisfactory, especially for those with malignant gliomas. Most low‐grade gliomas (LGGs) will finally develop into glioblastoma (GBM). GBM, the most common type, and malignant glioma have a median overall survival of only 15 months and a 5‐year survival rate of around 5%.^11^, ^12^

Increasing evidence indicates that immunotherapy has become an effective and promising therapeutic approach for gliomas.^13^, ^14^ Tumour‐associated macrophages (TAMs) are a critical component in the tumour microenvironment (TME) that contribute to tumour growth and metastasis by secreting various chemokines, cytokines and growth factors. TAMs induce the suppressive immune checkpoint factors to release from other immune cells in TME and, meanwhile, provide many immunotherapeutic blockade targets for inhibiting tumour progression.^15^ TAMS can be activated and polarized into two phenotypes, including classically activated (M1) and alternatively activated (M2) macrophages. M1 macrophages can produce pro‐inflammatory cytokines such as IL‐6, IL‐12 and TNF‐α were thought to exhibit anti‐tumour aspects.^16^, ^17^ On the contrary, M2‐like macrophage, secreting anti‐inflammatory cytokines like IL‐10, TGF‐β and arginase one, were associated with a poor prognosis through providing an immunosuppressive microenvironment to favour tumour growth.^18^, ^19^ However, the mechanisms of macrophage polarization in gliomas are still far from understanding.

This study selected upregulated and downregulated genes in distinguishing between M1 and M2 macrophage subtypes from GSEA‐MSIGDB. We found several genes associated with glioma prognosis and built a risk model based on the CGGA database. We also explored the relationship between the expression levels of these genes and immune infiltration. In addition, the reliability of this risk model was verified by the TCGA database and our sequencing data from gliomas.

## 2 MATERIALS AND METHODS

### 2.1 Samples and clinical data

We used clinical and RNA sequencing (RNA‐seq) data from CGGA (http://www.cgga.org.cn/download.jsp)^20^, ^21^ as our training cohort and data from TCGA (http://cancergenome.nih.gov) as our validating cohort. Meanwhile, human glioma samples were provided from the Clinical Diagnosis and Therapy Center for Glioma of Xiangya Hospital. A total of 24 clinical and RNA sequencing (RNA‐seq) data from adult glioma patients who underwent neurosurgery at the Department of Neurosurgery, Xiangya Hospital, were included in the study, of which 18 received TMZ treatment after surgery. Additional 10 clinical samples, including 5 LGGs and 5 GBMs were collected for q‐RT‐PCR. All glioma samples had clear pathological diagnoses and IDH1, MGMT and 1p/19q gene status. Informed consent of all subjects has been obtained in this study.

### 2.2 Gene signature building

The gene list was downloaded from the Human Molecular Signatures Database (GSEA‐MSIGDB) (http://www.gsea‐msigdb.org/gsea/msigdb/index.jsp). Genes with *P* values less than 0.1 were included by Univariate Cox analysis first. Multivariable Cox proportion hazard regression models were then built to investigate the relationship between risk scores with other clinical factors. Next, we grouped these samples into the low‐risk and high‐risk groups according to their risk score in the CGGA database. The risk score was expressed as gene 1 (expression × coefficient) + gene 2 (expression × coefficient) + gene 3 (expression × coefficient).

### 2.3 Related characteristics analysis

Go and KEGG pathway and protein–protein interaction (PPI) network analysis were performed by Metascape (https://metascape.org/gp/index.html#/main).^22^ The immune infiltrations were studied by Tumor Immune Estimation Resource (TIMER, https://cistrome.shinyapps.io/timer/)^23^ and Sangerbox (http://sangerbox.com/Index). We used the log‐rank test's Kaplan–Meier (KM) analysis to compare patients' overall survival (OS). R was used to study the low‐risk and high‐risk groups of clinical factors (grade, IDH mutant status, MGMT promoter methylation, glioma subtype and 1p19q codeletion).

### 2.4 RNA extraction and quantitative q‐RT‐ PCR analysis

To further validate the result of the macrophage polarization‐related gene signatures, q‐RT‐PCR was performed. miRNAs were extracted from the remaining biological replicates of LGGs (*n* = 5) and GBMs (*n* = 5) samples. A miRNA purification kit (Biosynthesis, Lewisville, TX) was used during this process. Subsequently, cDNA was synthesized for each sample using the miRNA First‐Strand synthesis kit following the manufacturer's instructions (Clontech, Mountain View, CA). The following primers were used: CD300C (forward: AGCGTGACCAGAAAGGACAGCC, reverse: GCTTCTCTGAGGTCTGTTCACC), CNRIP1 (forward: CGGTCTTTTACAAGGTGGACG, reverse: AGTTCCAGTGGGACAAGCACA), MYO1F (forward: CTTTGCCCGAACCATCCAGAA, reverse: CCGACGAAGTTCCGATTGATG), and β‐actin (forward: CACCATTGGCAATGAGCGGTTC, reverse: AGGTCTTTGCGGATGTCCACGT).

MiRNA primers were designed for the three selected genes (Integrated DNA technologies Inc., Coralville, IA). The miRNA expression of each gene was quantified using a SYBR q‐RT‐PCR kit (Clontech) and the cDNA from LGGs (*n* = 5) and GBMs (*n* = 5) samples. β‐Actin was used as a reference gene. Finally, the relative absolute expression of CD300C, CNRIP1 and MYO1F was calculated.

### 2.5 Statistical analysis

The Student's *t*‐test was applied to compare differences between groups, and ANOVA analysis was used in multiple groups comparing multivariate and univariate Cox regression analysis were performed to screen for variables of interest. A log‐rank test was performed to compare OS between different groups. Meanwhile, the Spearman test was used for correlation analysis. All statistical tests were two‐sided, and *p* < 0.05 was considered a significant difference. All analyses were performed under the R language.

## 3 RESULTS

### 3.1 The expression, enrichment pathways and PPI network of macrophage polarization‐related genes

**FIGURE 1:**
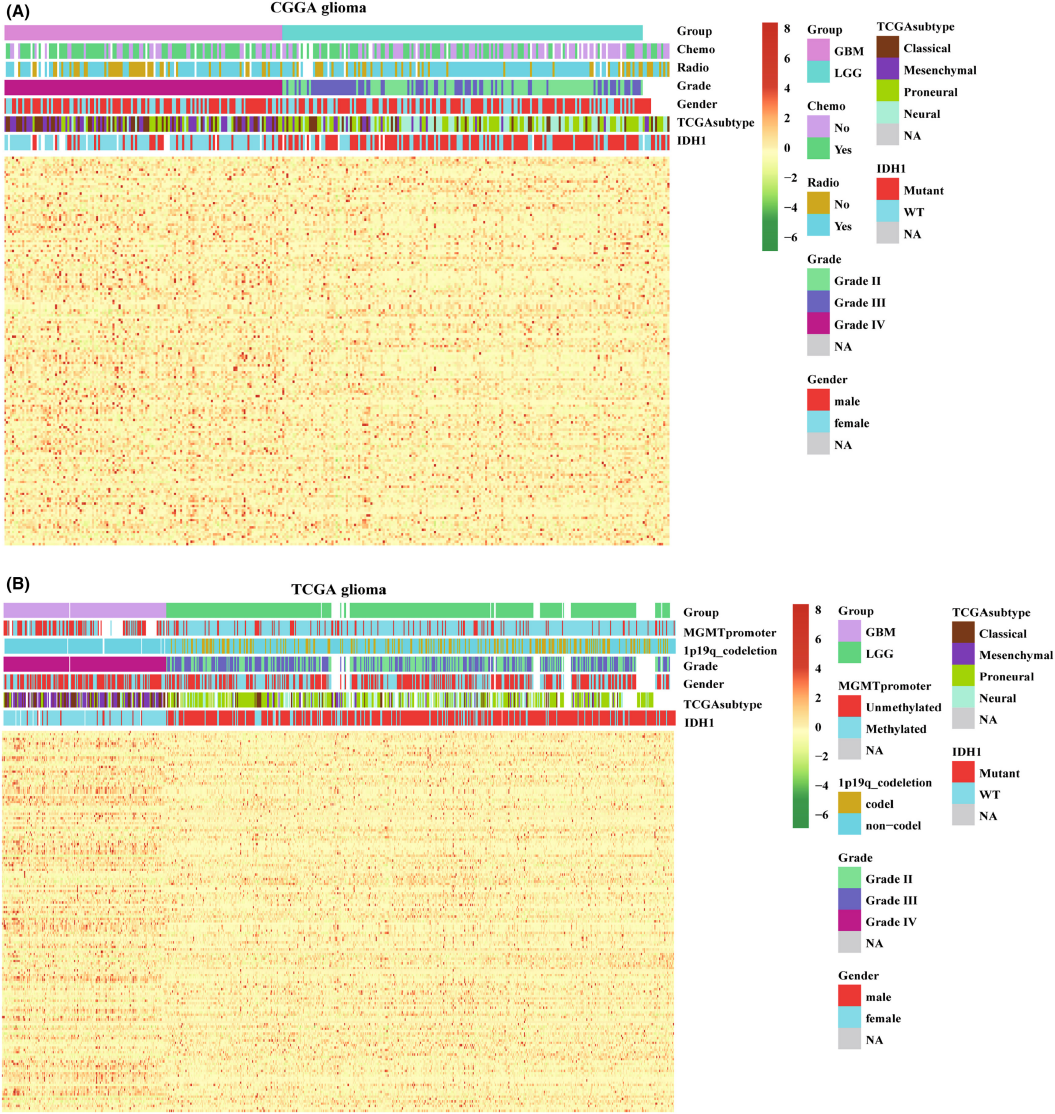
Expression of macrophage polarization‐related genes in gliomas. Correlation of macrophage polarization‐related genes expression and clinicopathologic characteristics in CGGA (A) and TCGA (B) databases.

**FIGURE 2:**
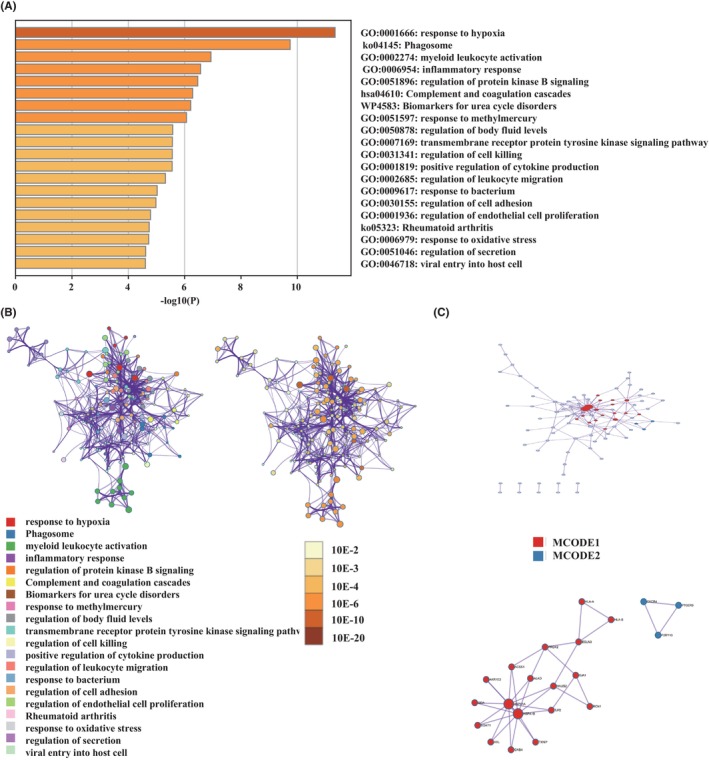
Enrichment pathways and PPI network of macrophage polarization‐related genes. Bar graph of top 20 enriched terms across macrophage polarization‐related genes (A). Network of enriched terms coloured by cluster‐ID and by *p*‐value (B). PPI network and MCODE components are identified in the gene lists (C).

The workflow of this article is depicted in Figure S1. First, a total of 170 genes that distinguish M1 and M2 macrophage was chosen from GSEA‐MSIGDB (Table S1). Second, to identify the relationship between the expression of macrophage polarization‐related genes and clinical characteristics in gliomas, we explored the RNA‐seq data of patients with gliomas from CGGA and TCGA databases. Both results in the CGGA database (Figure 1A) and TCGA database (Figure 1B) showed that most of these genes were upregulated in GBM compared to LGG. In addition, high levels of macrophage polarization‐related gene expression were associated with IDH1 WT status, 1p19q non‐codeletion, MGMT promoter unmethylation and were more common in classical and mesenchymal subtypes. GO and KEGG enrichment pathways analysis indicated various essential pathways correlated with these genes (Table S2). The top five enrichment pathways include hypoxia, phagosome, myeloid leukocyte activation, inflammatory response, protein kinase B signalling regulation, complement and coagulation cascades (Figure 2A,B). In addition, we performed a PPI enrichment analysis to determine the gene set enrichment terms (Figure 2C). The Molecular Complex Detection (MCODE) algorithm revealed that both signs of progress focus on response to oxidative stress, and antigen processing and presentation were involved in MCODE1. Meanwhile, the progress related to class A rhodopsin‐like GPCRs, G alpha (i) signalling events, and Class A/1 (rhodopsin‐like receptors) were involved in MCODE2 (Table S3).

### 3.2 Build a risk model based on the expression of macrophage polarization‐related genes in CGGA and verified in TCGA

**FIGURE 3:**
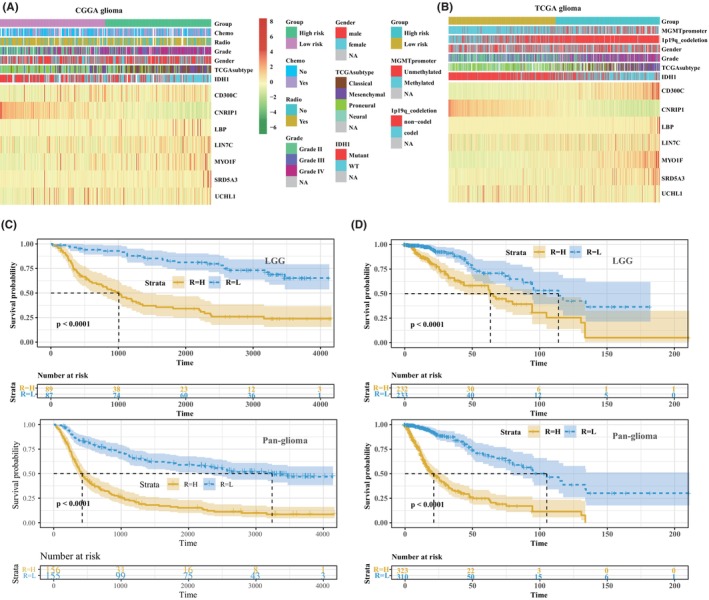
Expression of seven risk‐related genes and overall survival status of glioma patient based on risk score model in training and validating cohorts. Seven risk‐related genes were associated with clinicopathologic characteristics in CGGA (A) and TCGA (B) databases. Kaplan–Meier overall survival curve between high and low‐risk groups in LGG and pan‐gliomas patients from CGGA (C) and TCGA (D) databases.

Then, we build a gene‐based risk model from the CGGA database using univariate and multivariate regression analysis (Tables S4 and S5). Consequently, a seven‐gene signature was selected, and the risk score was as follows: (0.276 × LBP expression) + (−0.062 × CNRIP1 expression) + (0.002 × UCHL1 expression) + (−0.506 × CD300C expression) + (0.063 × MYO1F expression) + (0.083 × LIN7C expression) + (0.044 × SRD5A3 expression). Results showed that the high‐risk group was associated with high‐grade, IDH1 WT status and more prone to be classical and mesenchymal subtypes in the CCGA database (Figure 3A). Patients with LGG or pan‐glioma in high risk suffered an unfavourable prognosis (*p*<0.0001) compared to the low‐risk group (Figure 3C). We also verify the stability of this risk model in the TCGA database (Figure 3B,D). The same results were seen from the TCGA database; patients in the high‐risk group were associated with 1p19q non‐codeletion and MGMT unmethylation, along with a poor outcome (*p*<0.0001) compared to the low‐risk group.

### 3.3 The relationship between macrophage polarization‐related gene signature and clinicopathological characteristics

**FIGURE 4:**
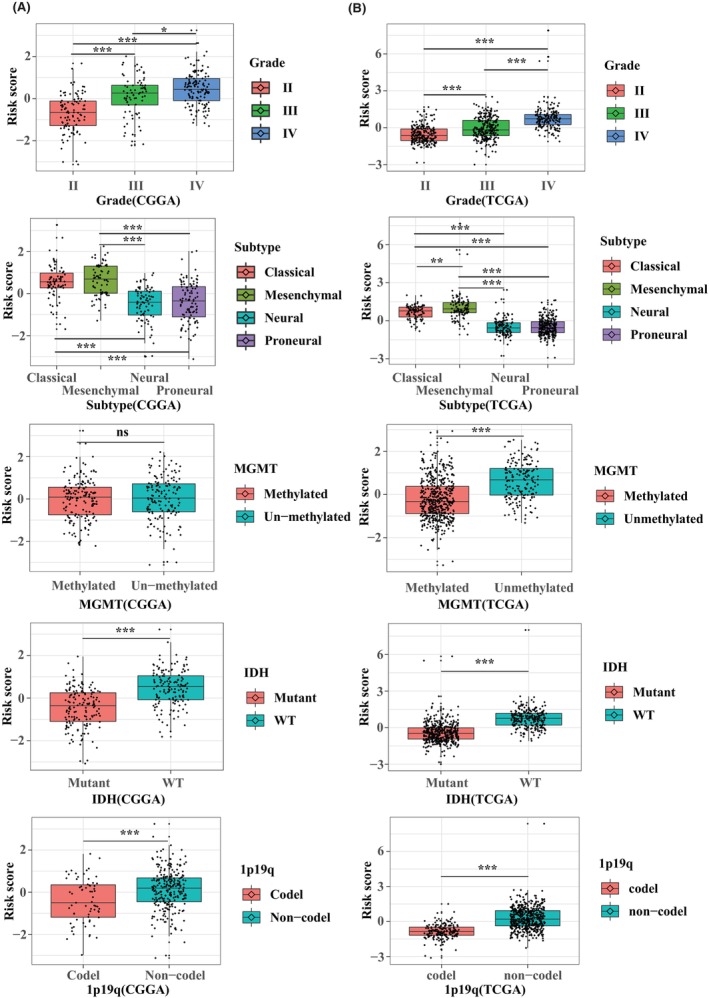
Correlation of risk score and clinicopathologic characteristics in training and validating cohorts. The relationship between risk score and gliomas grade, subtypes, MGMT promoter methylated status, IDH mutant status, 1p19q codeletion status in CGGA (A) and TCGA (B) databases. *p < 0.05, **p < 0.01, ***p < 0.001. ns, no significant differences.

**FIGURE 5:**
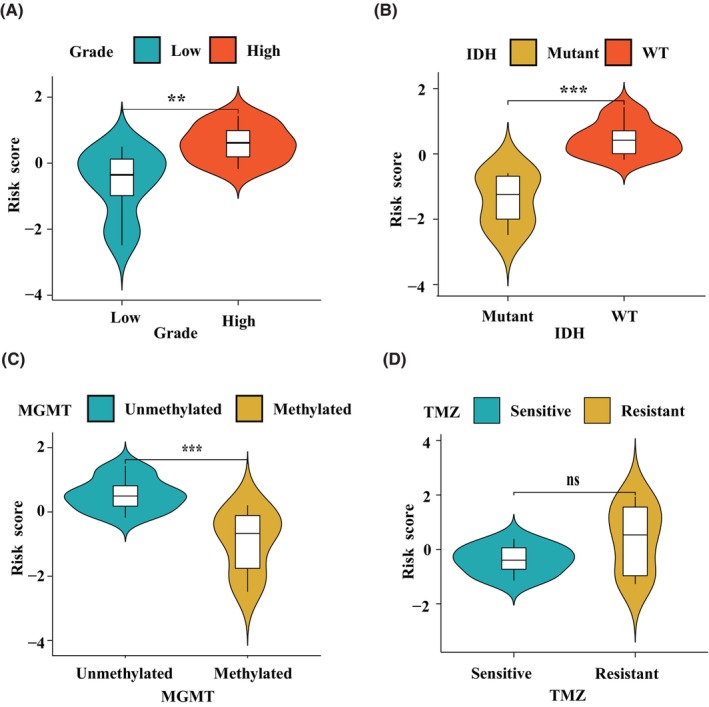
Correlation of risk score and clinicopathologic characteristics in our sequencing data. The relationship between risk score and gliomas grade, subtypes, MGMT promoter methylated status, IDH mutant status, TMZ sensitivity in our data (A–D). **p < 0.01, ***p < 0.001. ns, no significant differences.

Next, we studied the relationship between macrophage polarization‐related gene signatures and clinicopathological characteristics in the CGGA database (Figure 4A) and TCGA database (Figure 4B). Data showed that glioma patients with high‐risk scores were prone to have a high‐grade, classical and mesenchymal subtypes, IDH WT status and 1p19q non‐codeletion, which were consistent with the previous results heatmap. Data from TCGA also indicated that glioma patients with MGMT unmethylation have a high‐risk score. Moreover, we verified these results using our sequencing data (Figure 5). The high‐risk score was associated with high pathologic grade, IDH WT status and MGMT promoter unmethylation (Figure 5A–C). Glioma patients with TMZ resistance seem to have a high‐risk score even if the significance is above 0.05. This statistically non‐significant result might be due to the small sample size for adequate analysis.

### 3.4 The relationship between risk‐related gene expression and immune infiltration in the microenvironment of gliomas

**FIGURE 6:**
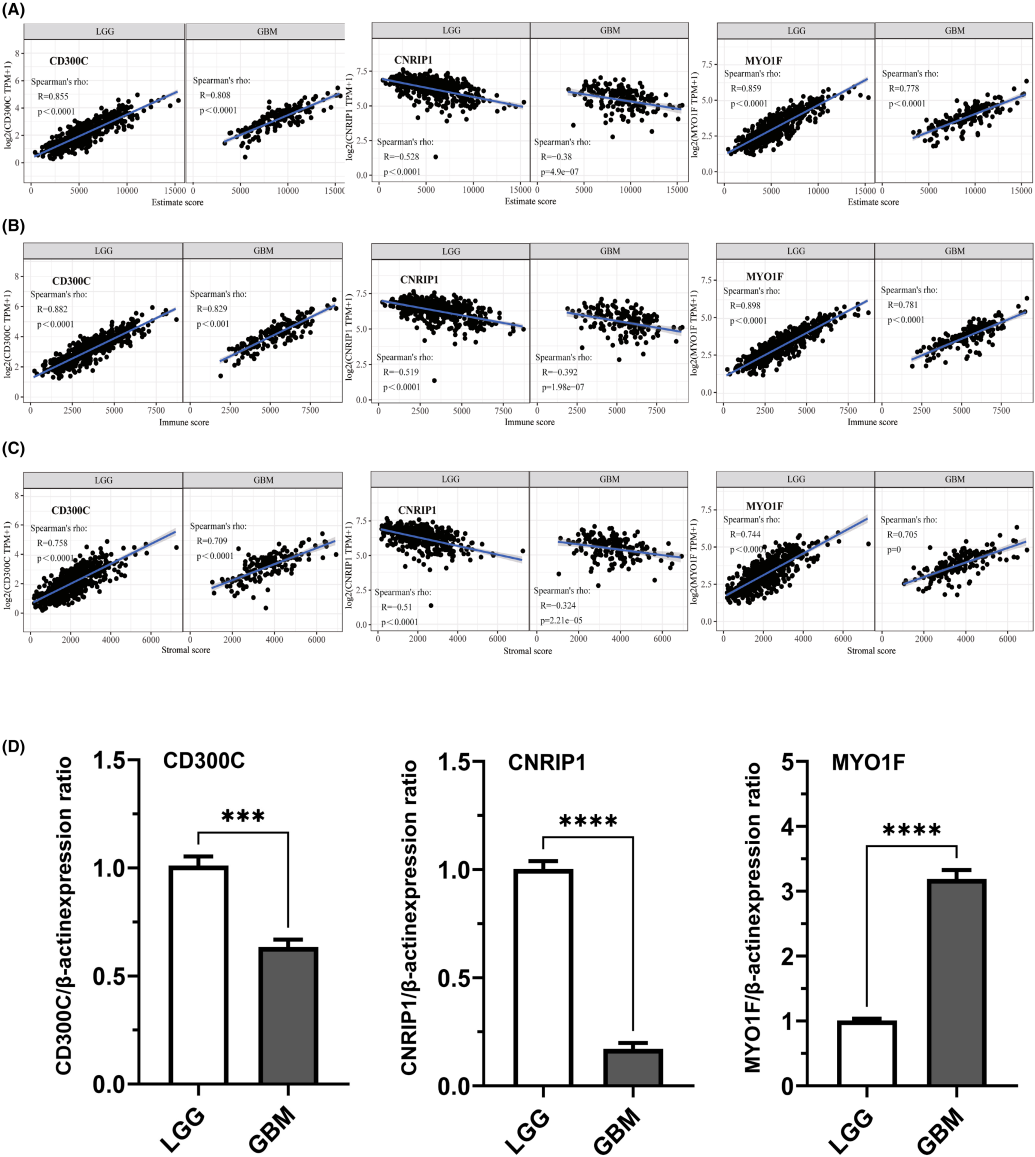
Correlation of risk‐related genes and estimate score, immune score, stromal score in gliomas. The relationship of CD300C, CNRIP1, MYO1F levels and estimate score (A), immune score (B), and stromal score (C) in gliomas microenvironment. Total RNAs were extracted from low‐grade glioma samples (*n* = 5) and glioblastoma (GBM) samples (*n* = 5). q‐RT‐PCR (D) was performed to test the target gene expression. The bar graph represents the relative expression of these genes. ***p ≤ 0.001, ****p ≤ 0.0001.

**FIGURE 7:**
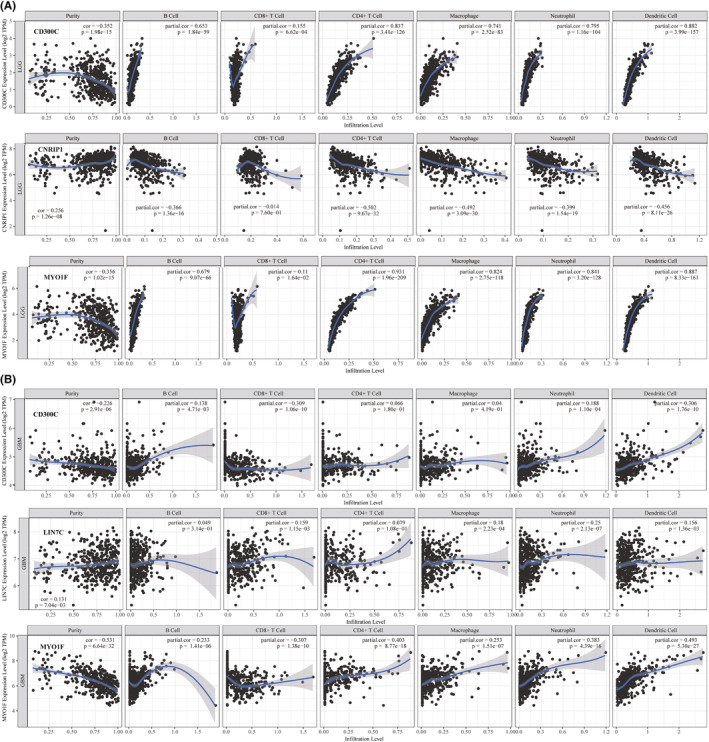
Correlation of risk‐related genes and immune cells infiltrations in LGG and GBM. The relationship of CD300C, CNRIP1, MYO1F levels and tumour purity, B cell, CD4+ T cell, CD8+ T cell, macrophage, neutrophil and dendritic cell infiltration in LGG (A) and GBM (B) microenvironment.

Finally, we performed GEPIA to study the relationship between risk‐related gene expression and immune infiltration in the microenvironment of gliomas. Results showed that the expression levels of these genes were correlated with estimate score, immune score and stromal score (Figure 6A–C and Figure S2). The top three most related genes are CD300C, CNRIP1 and MYO1F(*p*<0.0001). The differential expression of these genes was confirmed by q‐RT‐PCR results of additional 10 clinical samples (Figure 6D). The results showed a significant expression difference between LGGs and GBM, which indicate that these genes play an essential role in glioma immune microenvironments. We also explored the role of these genes in tumour purification and immune cell infiltration both in LGG and GBM (Figure 7 and Figure S3). Most of these genes were significantly associated with glioma purification. Meanwhile, CD300C, CNRIP1 and MYO1F were found to have a close relationship with B cells, CD4+ T cells, CD8+ T cells, macrophages, neutrophils and dendritic cell infiltration. Other genes also exhibit different abilities in inducing immune cell infiltration.

## 4 DISCUSSION

TAMs are known as a critical component in the tumour microenvironment, previous studies have tried to identify a macrophage‐related gene signature and initially revealed its prognostic significance and accuracy for glioma patients.^24^ In this study, we discussed the clinical prognosis and immune landscape of genes that distinguish M1 and M2 macrophages from CGGA and TCGA databases. The using of multilayer network approach to elucidate the interactions between tumour cell and tumour‐associated microenvironment has been proven to have an advantage in identifying prognostic and predictive signatures of cancer patients.^25^, ^26^ The results in this study indicated that most of these macrophage polarization‐related genes were associated with IDH1 WT status, 1p19q non‐codeletion and MGMT promoter unmethylation, and were more common in classical and mesenchymal subtypes. Meanwhile, enrichment pathways showed that multiple vital pathways involving tumour progress and immune response were associated with these genes, such as response to hypoxia, inflammatory response and immune cell activation. These results demonstrated that macrophage polarization plays a vital role in the malignancy of gliomas, which was consistent with previous studies.^27^, ^28^, ^29^

To improve the diagnosis and prognosis glioma patients, different types of risk model were built and tested based on genes/proteins biomarker signatures according to previous studies. Yin et al. reported that vesicle‐associated membrane protein 2 (VAMP2) and protein 5 (VAMP5) were identified as two SRGs affecting the prognoses of glioma patients in their risk model based on the key SNARE proteins.^30^ Zhou et al. developed a risk model based on telomere‐associated gene signatures to predict 1‐, 3‐, 5‐year overall survival, immune cell infiltration and drug sensitivity of glioma patients.^31^ Chen et al. developed a risk model based on six prognostic signatures comprising disulfidptosis‐related lncRNAs (DRLs).^32^ We build gene‐based risk model from the CGGA database, verified the reliability of this risk model in the TCGA database and calculated the risk score. Patients in the high‐risk group exhibited more malignant clinical features, including high pathologic grade, IDH WT status, MGMT promoter unmethylation and 1p19q non‐codeletion. They had a poor prognosis compared to the low‐risk group. More importantly, the reliability of these results was validated using our sequencing data again. In addition, we found that patients in the high‐risk model seem to suffer from TMZ resistance even with no significance. More samples need to be included to study the actual relationship between TMZ sensitivity and this risk model in the future.

With many immune checkpoints discovered, glioma immunotherapy has received great success in recent years.^33^, ^34^, ^35^ Immune checkpoint blockade targeting programmed cell death protein‐1 (PD‐1) in gliomas is a novel promising therapeutic strategy.^36^, ^37^, ^38^ M1 macrophage, with a pro‐inflammatory aspect, was an immune promoter in the tumour microenvironment that induced the immune system to clear tumour cells.^39^, ^40^ On the contrary, the M2 macrophage, with an anti‐inflammatory profile, was an immune suppressor in the tumour microenvironment that prevents tumour cells from being recognized by the inner immune system.^41^, ^42^, ^43^ A recent study found that tumour‐associated macrophages can drive immune checkpoint blockade resistance through CD4+ T cell suppression via PD‐1/PD‐L1 signalling.^44^ However, a single immune checkpoint often plays a limited role due to the heterogeneity of gliomas, and more immune checkpoints need to be found using high‐throughput sequencing.

This study established a seven‐genes‐based risk model and explored the relationship between gene levels and immune response. We found that these genes were tightly related to estimating score, immune score and stromal score. Meanwhile, they play an essential role in promoting immune cell infiltration, such as B cells, CD4+ T cells, CD8+ T cells, macrophages, neutrophils and dendritic cells. Especially, CD300C, CNRIP1 and MYO1F are the top three genes that engage in immune response in the glioma's microenvironment. CD300C, a member comes from the CD300 family, was found to play an important role in regulating the immune system.^45^, ^46^ The study indicated that the upregulated CD300C on M1 macrophage can negatively regulate CD4+ T cell and CD8+ T‐cell immunity.^46^, ^47^ Limit studies found that the overexpression of CNRIP1 can reduce the proliferative and migration abilities of colon cancer cells.^48^ Recent study reveals that Vav1‐MYO1F alters T‐cell differentiation and leads to accumulation of tumour‐associated macrophages in the tumour microenvironment, proven to be a feature linked with aggressiveness in human peripheral T‐cell lymphoma (PTCL).^49^ However, the exact role and mechanisms of these genes in macrophage polarization are still unknown.

There are several limitations to this study. First, our sequencing data sample size is too small to fully clarify the relationship between TMZ sensitivity and the risk model; more samples need to be included in the future. Second, we established a seven‐genes‐based risk model; the type of macrophages they expressed has not been studied. Meanwhile, the actual function and signalling pathways involved in macrophage polarization are not revealed in this study. Third, these genes play an essential role in immune response; their role in regulating immune infiltration has not been explored through in vivo and in vitro studies. Fortunately, our team has found a novel glioma model using patient‐derived glioma cerebral organoids and xenografts for disease modelling and drug screening.^50^ These limitations will be successfully solved in our future study.

## 5 CONCLUSION

In general, our study explored the expression and clinical features of macrophage polarization‐related genes in gliomas. It provided a promising prognostic predict risk model for patients with gliomas based on macrophage polarization‐related gene signature. This study will also contribute to fully clarify the fundamental mechanisms of macrophage polarization in the gliomas microenvironment and provide novel immunotherapy targets against gliomas in the future.

## AUTHOR CONTRIBUTIONS

**Weiming Zhong:** Conceptualization (lead); data curation (lead); formal analysis (lead); methodology (lead); software (supporting); writing – original draft (lead); writing – review and editing (lead). **Kaifen Xiong:** Data curation (supporting); Data analysis (supporting); visualization (supporting); review and editing (supporting). **Shuwang Li:** Data curation (supporting); investigation (supporting); methodology (supporting); software (supporting); validation (supporting). **Chuntao Li:** Conceptualization (supporting); project administration (lead); resources (equal); supervision (lead); writing – review and editing (supporting).

## FUNDING INFORMATION

The research was funded by the National Natural Science Foundation of China under grant no. 82001223, the National Natural Science Foundation of China under grant no. 81901401 and the Natural Science Foundation for Young Scientists of Hunan Province, China (grant no. 2019JJ50952).

## CONFLICT OF INTEREST STATEMENT

The authors declare that the research was conducted without any commercial or financial relationships that could be construed as a potential conflict of interest.

## Supporting information


**Figure S1.** Flow chart of the study design.

**Figure S2.** Correlation of risk‐related genes and estimate score, immune score, stromal score in gliomas. The relationship of LBP, LIN7C, SRD5A3, UCHL1 levels and estimate score (A), immune score (B), stromal score (C) in gliomas microenvironment.

**Figure S3.** Correlation of risk‐related genes and immune cells infiltrations in LGG and GBM. The relationship of LBP, LIN7C, SRD5A3, UCHL1 levels and tumour purity, B cell, CD4+ T cell, CD8+ T cell, macrophage, neutrophil and dendritic cell infiltration in LGG(A) and GBM(B) microenvironment.

**Table S1.** Genes distinguishing between M1 and M2.

**Table S2.** Pathways correlated with genes distinguishing between M1 and M2.

**Table S3.** Progress that involved in MCODE1 and MCODE2.

**Table S4.** Univariate and multivariate regression analysis of CGGA database.

**Table S5.** Univariate and multivariate regression analysis of TCGA database.

## Data Availability

The data that support the findings of this study are available from the corresponding author upon reasonable request. More data that supports the findings of this study are available in the supplementary material of this article.
