# Supplementary material for: Macrophage polarization‐related gene signature for risk stratification and prognosis of survival in gliomas
Source: J Cell Mol Med. 2024 Oct 24;28(20):e70000. doi: 10.1111/jcmm.70000 (PMC11502305; doi:10.1111/jcmm.70000)

Supplement **Figure 1**

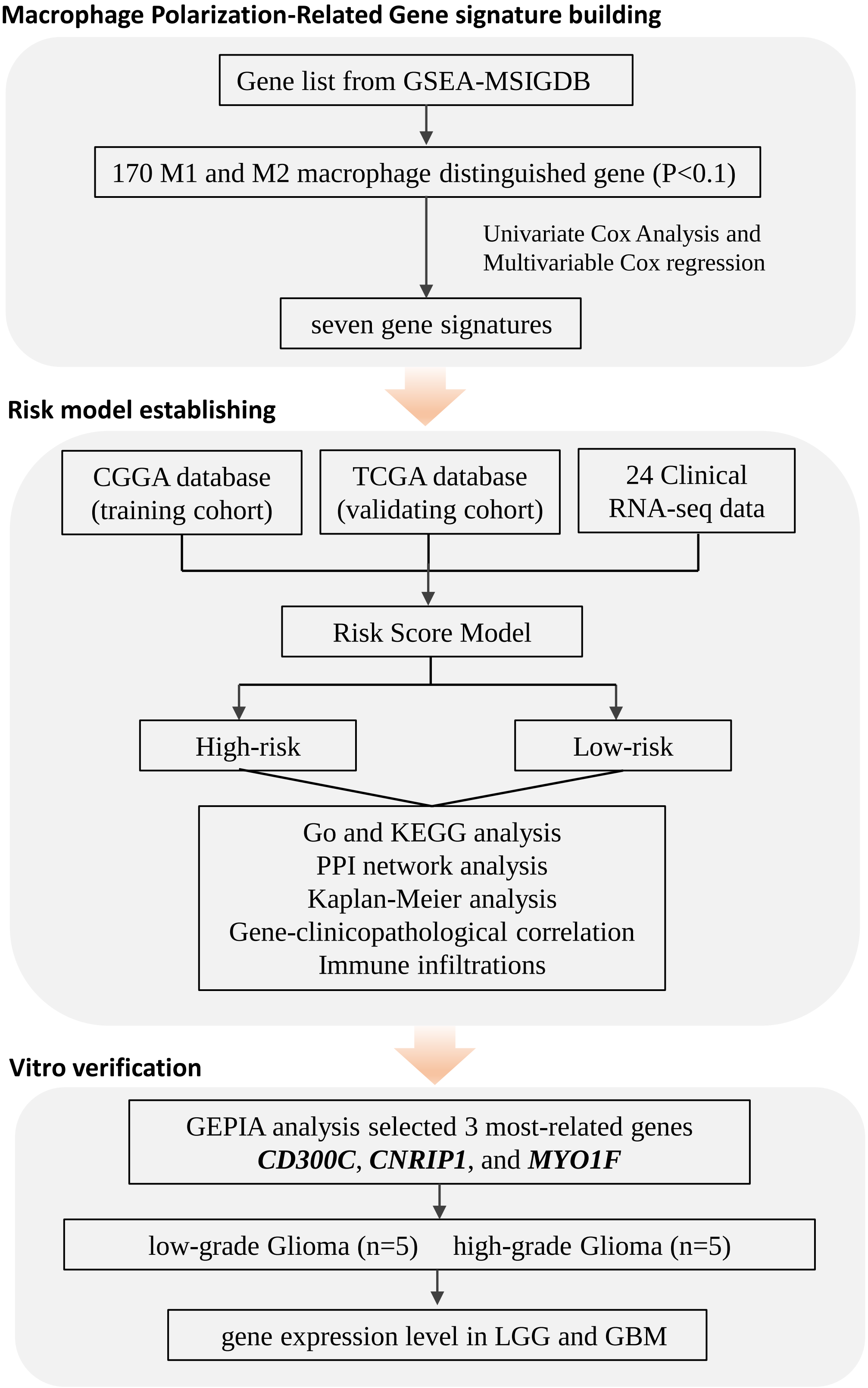

Supplement: Supplementary file 1 — Figure S1. Flow chart of the study design. [file JCMM-28-e70000-s004.pdf]
