# Supplementary figures and images for: Macrophage polarization‐related gene signature for risk stratification and prognosis of survival in gliomas
Source: J Cell Mol Med. 2024 Oct 24;28(20):e70000. doi: 10.1111/jcmm.70000 (PMC11502305; doi:10.1111/jcmm.70000)

# Supplement Figure 2

**A**

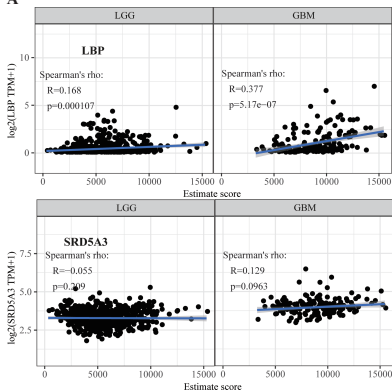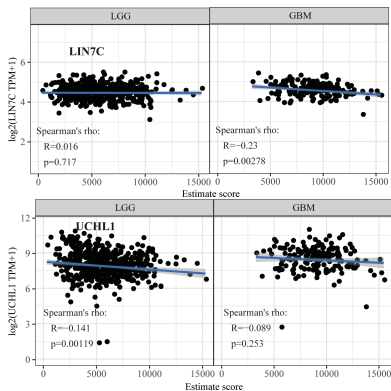

**B**

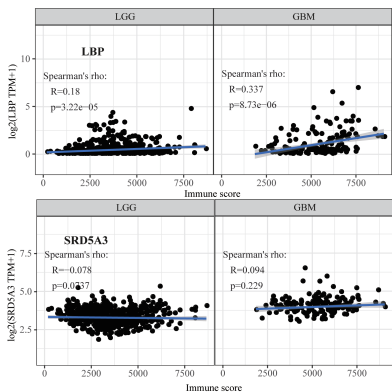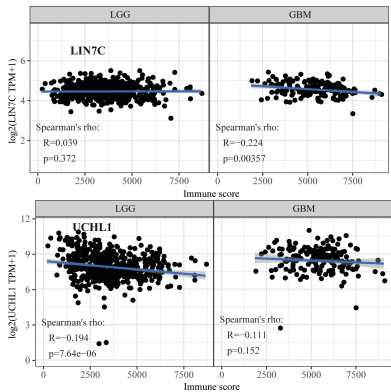

**C**

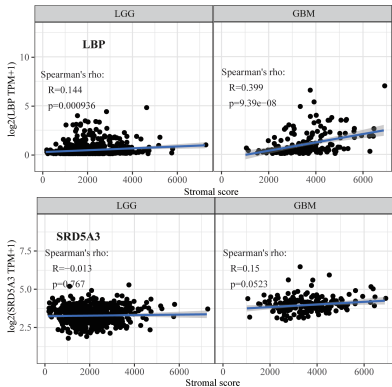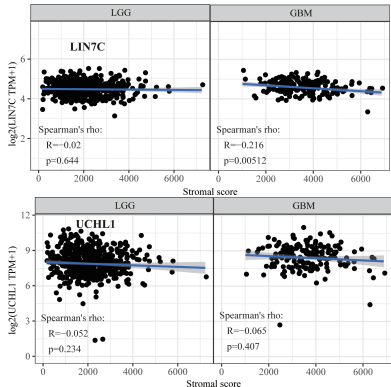

Supplement: Supplementary file 2 — Figure S2. Correlation of risk‐related genes and estimate score, immune score, stromal score in gliomas. The relationship of LBP, LIN7C, SRD5A3, UCHL1 levels and estimate score (A), immune score (B), stromal score (C) in gliomas microenvironment. [file JCMM-28-e70000-s005.pdf]

# Supplement Figure 3

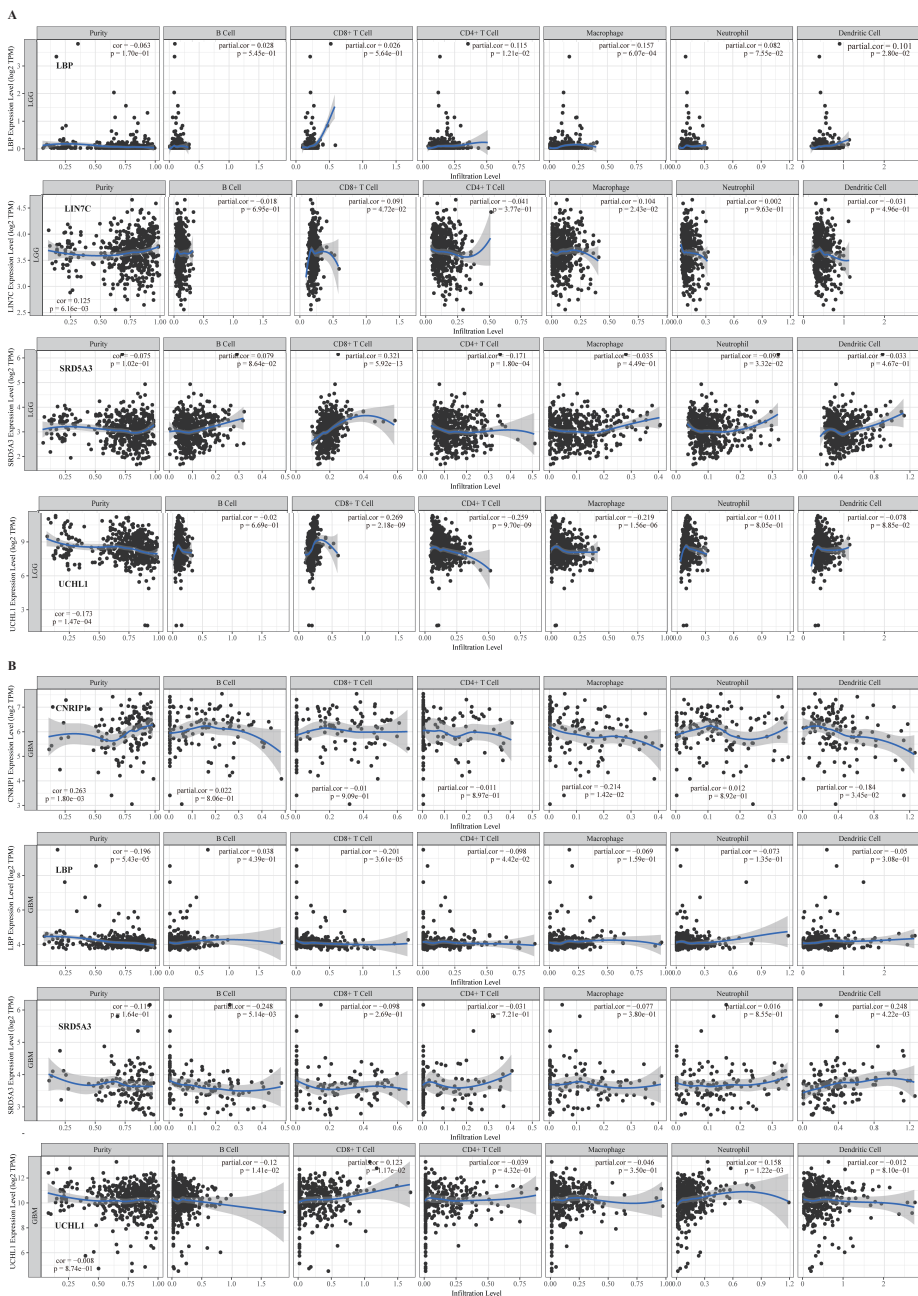

Supplement: Supplementary file 3 — Figure S3. Correlation of risk‐related genes and immune cells infiltrations in LGG and GBM. The relationship of LBP, LIN7C, SRD5A3, UCHL1 levels and tumour purity, B cell, CD4+ T cell, CD8+ T cell, macrophage, neutrophil and dendritic cell infiltration in LGG(A) and GBM(B) microenvironment. [file JCMM-28-e70000-s003.pdf]
