# Supplementary material for: Macrophage polarization‐related gene signature for risk stratification and prognosis of survival in gliomas
Source: J Cell Mol Med. 2024 Oct 24;28(20):e70000. doi: 10.1111/jcmm.70000 (PMC11502305; doi:10.1111/jcmm.70000)
Supplement: Supplementary file 4 — Table S1. Genes distinguishing between M1 and M2. [file JCMM-28-e70000-s001.docx]

Supplement Table 1

Genes distinguishing between M1 and M2

| Up-regulated genes | Down-regulated genes |
| --- | --- |
| ABCA9 | ADAM8 |
| ABHD1 | AK4 |
| ACSS1 | AKR1E2 |
| ACYP2 | ALDOC |
| ADA | ANG |
| AIF1 | ANKRD37 |
| ALAD | ANOS1 |
| ARFGEF3 | ARG1 |
| ARSB | ARSK |
| ATP6V0E2 | ATG4D |
| AXL | ATP6V0D2 |
| BCKDHB | BNIP3 |
| BLOC1S6 | BST1 |
| CADM1 | C1QB |
| CAP1 | C1QC |
| CAPN5 | C1QTNF12 |
| CBX6 | C5orf34 |
| CD59 | CBLB |
| CFH | CD24 |
| CLBA1 | CD300C |
| CNRIP1 | CD300LD |
| COLEC12 | CD5L |
| COMT | CHIA |
| CRIM1 | COL20A1 |
| CXCL14 | CRIPT |
| CXCR4 | CTSK |
| DST | CTSL |
| DYNLT1 | CYTIP |
| EMC1 | EFHD2 |
| ENO2 | EGLN3 |
| FAM124A | ERO1A |
| FAM135A | F10 |
| FAM9B | F7 |
| FGD2 | FAM177A1 |
| FILIP1L | FAM199X |
| GALNT11 | FAM241A |
| GATM | FBLIM1 |
| GDA | FLRT2 |
| GJA1 | GASK1B |
| GLO1 | GDF15 |
| GNB4 | GPRC5B |
| HAUS2 | HILPDA |
| HDDC3 | HLA-A |
| HLA-DQA1 | HLA-B |
| HMGN3 | HS6ST1 |
| KCNJ10 | HSPA1A |
| LAMA3 | HSPA1B |
| LCORL | IGF2R |
| LYPLAL1 | IGHM |
| MAF | IL18BP |
| MALAT1 | ITGB3 |
| MARCKSL1 | LBP |
| MARCO | LIN7C |
| MSR1 | LRRC27 |
| NAT8L | MCOLN3 |
| NRCAM | MFGE8 |
| OCEL1 | MGST2 |
| OGFRL1 | MYO1F |
| P2RY13 | PADI4 |
| PIANP | PDXDC1 |
| PIK3AP1 | PINK1 |
| PLAAT3 | PKDCC |
| PLBD1 | PRDX2 |
| PLXDC2 | PROCR |
| PPP2R5C | PTGER2 |
| PTGER3 | QRICH1 |
| RAB10 | RGCC |
| RAPSN | RIMBP2 |
| RASAL2 | RPGRIP1 |
| RCBTB2 | RRAS2 |
| RCN1 | SCD |
| RFX3 | SH2B2 |
| RPL14 | SLC2A1 |
| SFI1 | SNHG6 |
| SLC35A1 | SOAT1 |
| SLC7A7 | THBS1 |
| SLCO2B1 | TJP2 |
| SRD5A3 | TLR1 |
| TGFBI | TMEM267 |
| TIFAB | TULP4 |
| TM7SF3 | UCHL1 |
| TOR3A | VEGFA |
| TTC3 | XDH |
| TUBB2B |  |
| TXNIP |  |
| ZNF727 |  |
