# Supplementary material for: Macrophage polarization‐related gene signature for risk stratification and prognosis of survival in gliomas
Source: J Cell Mol Med. 2024 Oct 24;28(20):e70000. doi: 10.1111/jcmm.70000 (PMC11502305; doi:10.1111/jcmm.70000)
Supplement: Supplementary file 5 — Table S2. Pathways correlated with genes distinguishing between M1 and M2. [file JCMM-28-e70000-s002.docx]

**Supplement Table 2. Pathways correlated with Genes distinguishing between M1 and M2**

| GO | Category | Description | Count | % | Log10(P) | Log10(q) |
| --- | --- | --- | --- | --- | --- | --- |
| GO:0001666 | GO Biological Processes | response to hypoxia | 18 | 10.59 | -11.34 | -7.03 |
| ko04145 | KEGG Pathway | Phagosome | 12 | 7.06 | -9.74 | -5.99 |
| GO:0002274 | GO Biological Processes | myeloid leukocyte activation | 18 | 10.59 | -6.93 | -3.35 |
| GO:0006954 | GO Biological Processes | inflammatory response | 19 | 11.18 | -6.57 | -3.11 |
| GO:0051896 | GO Biological Processes | regulation of protein kinase B signaling | 11 | 6.47 | -6.47 | -3.06 |
| hsa04610 | KEGG Pathway | Complement and coagulation cascades | 7 | 4.12 | -6.28 | -2.99 |
| WP4583 | Wiki Pathways | Biomarkers for urea cycle disorders | 4 | 2.35 | -6.21 | -2.97 |
| GO:0051597 | GO Biological Processes | response to methylmercury | 3 | 1.76 | -6.07 | -2.88 |
| GO:0050878 | GO Biological Processes | regulation of body fluid levels | 14 | 8.24 | -5.58 | -2.66 |
| GO:0007169 | GO Biological Processes | transmembrane receptor protein tyrosine kinase signaling pathway | 17 | 10 | -5.56 | -2.66 |
| GO:0031341 | GO Biological Processes | regulation of cell killing | 7 | 4.12 | -5.56 | -2.66 |
| GO:0001819 | GO Biological Processes | positive regulation of cytokine production | 13 | 7.65 | -5.56 | -2.66 |
| GO:0002685 | GO Biological Processes | regulation of leukocyte migration | 9 | 5.29 | -5.32 | -2.44 |
| GO:0009617 | GO Biological Processes | response to bacterium | 16 | 9.41 | -5.02 | -2.28 |
| GO:0030155 | GO Biological Processes | regulation of cell adhesion | 16 | 9.41 | -4.98 | -2.26 |
| GO:0001936 | GO Biological Processes | regulation of endothelial cell proliferation | 8 | 4.71 | -4.79 | -2.14 |
| ko05323 | KEGG Pathway | Rheumatoid arthritis | 6 | 3.53 | -4.74 | -2.13 |
| GO:0006979 | GO Biological Processes | response to oxidative stress | 12 | 7.06 | -4.72 | -2.13 |
| GO:0051046 | GO Biological Processes | regulation of secretion | 14 | 8.24 | -4.62 | -2.06 |
| GO:0046718 | GO Biological Processes | viral entry into host cell | 7 | 4.12 | -4.61 | -2.06 |
