# Supplementary material for: Macrophage polarization‐related gene signature for risk stratification and prognosis of survival in gliomas
Source: J Cell Mol Med. 2024 Oct 24;28(20):e70000. doi: 10.1111/jcmm.70000 (PMC11502305; doi:10.1111/jcmm.70000)
Supplement: Supplementary file 6 — Table S3. Progress that involved in MCODE1 and MCODE2. [file JCMM-28-e70000-s008.docx]

**Supplement Table 3. Progress that involved in MCODE1 and MCODE2**

| MCODE | GO | Description | Log10(P) |
| --- | --- | --- | --- |
| MCODE_1 | ko04612 | Antigen processing and presentation | -6.8 |
| MCODE_1 | hsa04612 | Antigen processing and presentation | -6.8 |
| MCODE_1 | GO:0006979 | response to oxidative stress | -6.6 |
| MCODE_2 | WP455 | GPCRs, Class A Rhodopsin-like | -6.1 |
| MCODE_2 | R-HSA-418594 | G alpha (i) signaling events | -5.8 |
| MCODE_2 | R-HSA-373076 | Class A/1 (Rhodopsin-like receptors) | -5.8 |
