# Supplementary material for: Macrophage polarization‐related gene signature for risk stratification and prognosis of survival in gliomas
Source: J Cell Mol Med. 2024 Oct 24;28(20):e70000. doi: 10.1111/jcmm.70000 (PMC11502305; doi:10.1111/jcmm.70000)
Supplement: Supplementary file 7 — Table S4. Univariate and multivariate regression analysis of CGGA database. [file JCMM-28-e70000-s007.docx]

**Supplement Table 4. Univariate and multivariate regression analysis of CGGA database**

| CGGA cohort | | | | | | |
| --- | --- | --- | --- | --- | --- | --- |
|  | Univariate analysis | | | Multivariate analysis | | |
| Characteristics | P‐value | HR | 95% CI | P‐value | HR | 95% CI |
| Age | <0.001 | 1.033 | 1.020-1.047 | 0.669 | 0.997 | 0.981-1.013 |
| Gender | 0.621 | 1.074 | 0.808-1.428 | 0.639 | 1.081 | 0.781-1.496 |
| Grade | <0.001 | 2.692 | 2.224-3.259 | <0.001 | 2.267 | 1.334-3.838 |
| Subtype | <0.001 | 0.424 | 0.290-0.622 | 0.973 | 0.734 | 0.452-1.196 |
| IDH1 | <0.001 | 0.367 | 0.270-0.501 | 0.256 | 0.773 | 0.496-1.206 |
| Radio | <0.001 | 0.505 | 0.368-0.694 | 0.004 | 0.584 | 0.405-0.844 |
| Chemo | 0.001 | 1.658 | 1.219-2.255 | 0.146 | 0.757 | 0.521-1.102 |
| Risk score | <0.001 | 3.294 | 2.684-4.044 | <0.001 | 2.808 | 2.118-3.722 |
