# Supplementary material for: Macrophage polarization‐related gene signature for risk stratification and prognosis of survival in gliomas
Source: J Cell Mol Med. 2024 Oct 24;28(20):e70000. doi: 10.1111/jcmm.70000 (PMC11502305; doi:10.1111/jcmm.70000)
Supplement: Supplementary file 8 — Table S5. Univariate and multivariate regression analysis of TCGA database. [file JCMM-28-e70000-s006.docx]

**Supplement Table 5. Univariate and multivariate regression analysis of TCGA database**

| TCGA cohort | | | | | | |
| --- | --- | --- | --- | --- | --- | --- |
|  | Univariate analysis | | | Multivariate analysis | | |
| Characteristics | P‐value | HR | 95% CI | P‐value | HR | 95% CI |
| Age | <0.001 | 1.073 | 1.061-1.084 | <0.001 | 1.064 | 1.047-1.081 |
| Gender | 0.462 | 1.111 | 0.840-1.469 | 0.601 | 1.105 | 0.760-1.605 |
| Grade | <0.001 | 11.002 | 6.923-17.485 | <0.001 | 4.416 | 1.908-10.642 |
| Subtype | <0.001 | 4.154 | 2.793-6.185 | 0.097 | 1.031 | 0.539-1.975 |
| IDH | <0.001 | 0.117 | 0.085-0.161 | 0.164 | 0.576 | 0.264-1.253 |
| MGMT promoter | <0.001 | 0.337 | 0.247-0.461 | 0.435 | 0.838 | 0.538-1.306 |
| 1p/19q | <0.001 | 4.383 | 2.662-7.216 | 0.03 | 1.973 | 1.067-3.647 |
| Risk score | <0.001 | 1.009 | 1.007-1.010 | 0.115 | 1.003 | 0.999-1.008 |
